# Risk of second breast cancer in female Hodgkin’s lymphoma survivors: a meta-analysis

**DOI:** 10.1186/1471-2407-12-197

**Published:** 2012-05-28

**Authors:** Ezzeldin M Ibrahim, Khaled M Abouelkhair, Ghieth A Kazkaz, Osama A Elmasri, Meteb Al-Foheidi

**Affiliations:** 1 Oncology Center of Excellence, International Medical Center, PO Box 2172, Jeddah, 21451, Saudi Arabia; 2 Princess Noorah Oncology Center, Abdulaziz Medical City, P.O.BOX 9515, Jeddah, 21423, Saudi Arabia

## Abstract

**Background:**

Women treated for Hodgkin’s lymphoma (HL) have an elevated risk of developing second breast cancer (SBC) compared with the general population. We planned this meta-analysis to quantify the long-term risk of SBC and analyze the contributing risk factors among HL survivors.

**Methods:**

According to predefined selection criteria, literature search identified 34 studies that were included in the analyses.

**Results:**

After eliminating overlapping or duplicate data, 957 incidences of SBC were encountered in 24,505 females with HL over a median follow-up of 14.9 years. The medians: age at the diagnosis of HL, age at diagnosis of SBC, and latency since HL treatment to the development of SBC were 23.7, 35.0, and 17.7 years, respectively. The pooled relative risk (RR) of SBC was 8.23 (95% CI, 5.43-12.47, *I*^*2*^ = 96%), with a median absolute excess rate of 22.9 per 10,000 person-years. The RR was found inversely related to age at diagnosis of HL with the highest rate (68.7; [95%CI, 28.08-168.11], *I*^*2*^ = 79%), occurred in young patients (≤ 15 years old), where the RR in older women (≥ 40 years old) was not significant (0.55; [95% CI, 0.09-3.52]). Analysis of RR by 5-year increments since the treatment of HL showed that the risk was highest after 15–19 years of latency (13.87; [95% CI, 7.91-24.30], *I*^*2*^ = 89%). Analysis of the effect of treatment modalities showed that the RR rates were (4.70; [95% CI, 3.28-6.75], *I*^*2*^ = 74%), (5.65; [95%CI, 2.94-10.88], *I*^*2*^ = 91%), and (1.19; [95% CI, 0.50-2.82], *I*^*2*^ = 65%), for radiotherapy (RT) only, combined RT and chemotherapy (CT), and CT only, respectively. To investigate the demonstrated heterogeneity, meta-regression analysis was performed when feasible. In most such analyses, the natural logarithm of RR was inversely associated with age at HL diagnosis.

**Conclusions:**

We conclude that, the current meta-analysis provided the most recent comprehensive estimate of the risk of SBC in a broad-range of HL survivors. Younger age at diagnosis proved to be a dominant risk factor. The obtained results would serve providing breast cancer screening recommendations for HL survivors.

## Background

Hodgkin’s lymphoma (HL) became a curable disease by radiation therapy (RT) and/or combination chemotherapy (CT) since the early 70s [1-3]. Long-term disease-free survival of 70% to 90%, depending on stage at diagnosis has been achieved [4], and even more favorable outcome has been demonstrated in pediatrics, with a 5-year survival exceeding 90% [5].

However, increased risk of second cancer following effective treatment of HL has long been reported [6]. More recently, second neoplasms after HL are being encountered with increasing frequency due to the marked improvement in survival [2,7]. The particular elevated risk of second breast cancer (SBC) among this population is not surprising in view of the reported excess risks of breast cancer (BC) after incidental low doses of ionizing radiation [8,9], therapeutic RT [10,11], or as sequelae of the carcinogenic effects of CT [12,13].

Two pertinent meta-analyses have been published and they have addressed different questions. The first meta-analysis was published in 2006 and examined all second malignancy risk associated with HL treatment in 31 randomized trials and it included 65 incidence of SBC [14]. In the second meta-analysis [15], SBC risk and BC surveillance were investigated in young females (≤ 30 years at the primary tumor diagnosis) receiving moderate to high doses of RT targeted to mantle and modified mantle fields, mediastinum, lung, and thorax [15]. The latter meta-analysis comprised 11 studies that were not restricted to patients with HL but included all primary neoplasms in that age group.

Research on the late consequences of HL has often been limited by the size and composition of the study populations and by the duration and completeness of patient follow-up. To the best of our knowledge, there is no recently published meta-analysis intended to examine the risk of SBC in a broad range of ages at HL diagnosis, various follow-up periods, and subsequent to different therapeutic modalities. Also not precisely known, is the effect of other contributing risk factors. The lack of such data has prompted the current meta-analysis.

## Methods

### Search strategy

Between January 1966 and October 2011, we identified studies of interest by first conducting an electronic literature search of the databases MEDLINE, EMBASE, and the Cochrane Library. We also searched for relevant abstracts in the annual conference proceedings between January 1984 to October 2011 for the American Society of Clinical Oncology, European Society for Medical Oncology, and the San Antonio Breast Cancer Symposium. All ages of HL patients were eligible for inclusion.

We used exploded Medical Subject Heading terms or key words terms ‘lymphoma’, ‘Hodgkin’, ‘Hodgkin’s disease’ and ‘Hodgkin’s lymphoma’. The terms were combined with ‘neoplasm, second neoplasm, second primary’ using the Boolean operator ‘and’. Search results were also filtered against the terms ‘breast, breast cancer, breast neoplasm). In the second step, these keywords were combined using the Boolean operator ‘and’ with ‘standardized incidence ratio’, ‘relative risk’, and ‘observed to expected’. In addition, we manually reviewed the reference lists of relevant studies to identify additional pertinent published articles.

### Selection criteria

We included studies that met each of following criteria: (i) published in English language between January 1985 and October 2011; (ii) included naive patients at any age and with any stage of HL; (iii) investigated the risk for second malignant neoplasms (SMNs) in HL survivors; (iv) reported relative risk (RR) and/or specified as standardized incidence ratios (SIR) or data allowing such outcomes to be derived; and (v) published as original articles (no case reports, case series, reviews, comments, letters, or editorials).

When two or more references reported duplicate data, we only included in the analysis the most recent data, studies with the longer follow-up, or the most relevant studies. We excluded studies that mainly addressed the clinical characteristics of SBC. We also excluded studies that mainly intended to evaluate the potential benefits and harms associated with breast cancer surveillance among women with HL. Case–control designs, i.e. HL patients who developed BC compared with patients who did not were excluded.

### Data extraction

Two authors (KMA, and GAK) independently inspected each reference title identified by the search and applied the inclusion criteria. For possibly relevant articles and in cases of disagreement between reviewers, the full article was obtained and inspected independently by the five authors. The data intended for extraction were discussed, and decisions were documented. We used the STROBE (Strengthening the Reporting of Observational Studies in Epidemiology) reporting criteria to assess the quality of studies included in the meta-analysis [16]. Any significant lack of concordance in the scores assigned by authors was discussed to reach a consensus.

Standardized Excel sheet was used for each study that fulfilled the inclusion criteria. Extracted data included paper characteristics (first author’s last name, publication year, country in which the study was carried out, and data source), study design, number of HL patients, mean/median age of patients, mean/median duration of follow-up, therapy details, number of observed and expected SBC cases, and RR or standardized-incidence rate (SIR) with corresponding 95% confidence interval (CI). The ratio of observed to expected numbers of cancers, SIR (referred to in the text as RR) was then used or calculated with likelihood-based 95% CI from Poisson models [17]. Where not reported, we computed the CI for the risk assuming a Poisson distribution for the observed number of cases. Standard error (SE) for the natural logarithm of RR (lnRR) was derived from CI, applying the following equation: SE = ln(upper 95% CI/lower 95% CI)/(2 x z_1__- *a*/2_). When appropriate, we also used the built-in calculator of the Review Manager Software (version 5.1.4 for Windows; The Cochrane Collaboration, Oxford, UK) to compute missing data. When studies showed that the observed number of cases was zero, we simply added 1 to both the observed and the expected number of cases to allow computation of an estimate of the lnRR and its associated SE [18].

### Outcome measures

The primary outcome was the overall pooled RR of incidence of SBC among women survivors of HD. The secondary end points were RR vs. various variables: source of data, age at diagnosis of HD, length of follow-up, treatment modalities, and any additional relevant risk factors. RT in this meta-analysis is referred to supra-diaphragmatic irradiation with or without other radiation fields.

### Statistical analyses

We assessed heterogeneity of the studies’ results by inspecting graphical presentations and by calculating an *x*^2^ test of heterogeneity and the *I*^*2*^ statistic of inconsistency [19,20]. Statistically significant heterogeneity was defined as a *x*2 *P* value less than .1 or an *I*2 statistic greater than 50%. The estimates of RR, together with associated 95% CI, were obtained using the DerSimonian and Laird random-effects model [21]. Meta-regression analysis was performed to determine to what extent the heterogeneity is explained by various covariates using IBM SPSS statistical package v.19. The dependent variable was the lnRR weighted for the inverse of variance to perform weighted least square linear regression. We first conducted a univariate regression analysis for each variable followed by a multivariate regression including variables found significant in the univariate analysis.

Subgroup analyses were performed to assess potential contributions of various clinical variables to outcomes. A funnel plot estimating the precision of trials (plots of logarithm of the RR against the sample size) was examined for asymmetry to estimate publication bias [22]. Publication bias was also quantified by the regression asymmetry test by Egger [22].

All statistical tests were two-sided. RR was estimated according to the inverse of variance method with the use of Review Manager Software v5.1.4.

## Results

### Search results

**Figure 1:**
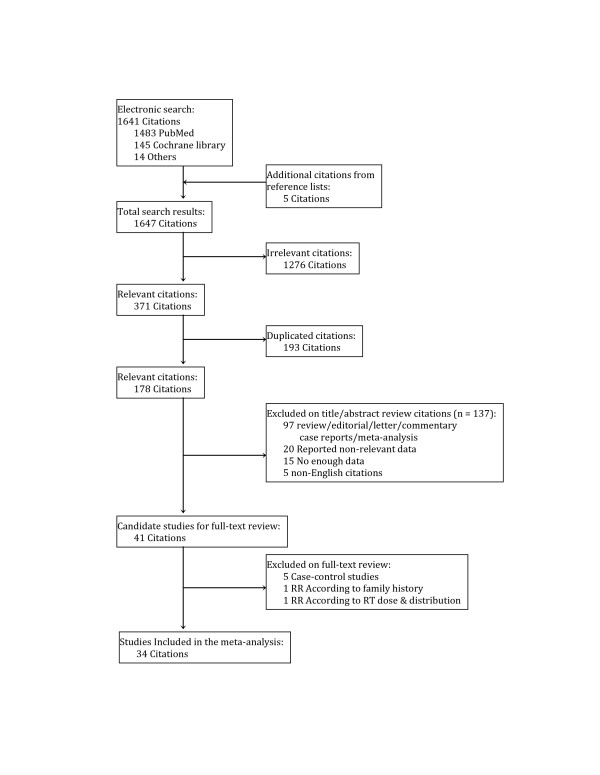
Search results and the selection of 33 included studies.

We identified 1,647 potentially relevant articles (Figure 1). After exclusion of duplicate references, none-relevant literature, and those that did not satisfy inclusion criteria, 41 candidate articles were considered for the meta-analysis [23-62]. After careful review of the full text of these articles, 7 studies were excluded. In 5 studies the RR was reported based on case–control design, i.e. HL patients who developed SBC against patients who did not [33,43,47,63,64]. The RR in the sixth excluded study compared RR among HL patients according to presence or absence of a family history of BC [50]. The seventh excluded study was designed to examine RT dose and dose distribution in 41 HL patients (25 females) treated at a single Canadian institution [49].

The remaining 34 studies were included in the meta-analysis. The funnel plot of 25 non-overlapping studies showed mild asymmetry, however, the Egger linear regression test was not significant (*P* = .059).

Of the 34 included studies, there were several reports of overlapping and/or updated data with longer follow-up and more encountered events. For any analysis, only the updated results were used unless there were relevant data available in earlier report and were not included in more recent publication. That approach was used to deal with the overlapping data of van Leeuwen et al. [25] (2 Dutch institutes) and De Bruin et al. [55] (5 Dutch institutes); Mauch et al. [26] (5 USA institutes) and Ng et al. [41] (4 USA institutes); Aisenberg et al. [28] and Alm El-Din et al. [54] (single USA institute); Basu et al. [52] and Constine et al. [53] (5 USA institutes); Neglia et al. [39], Kenney et al. [45], and Castellino et al. [59] (the multi-institutional USA Childhood Cancer Survivor Study); Metayer et al. [35] (16 population-based cancer registries in North America and Europe, Hodgson et al. [48] (13 population-based cancer registries in North America and Europe), and that of Dores et al. [57] (9 population-based cancer registries in the USA); Hancock et al. [24], Wolden et al. (1998) [30], O'Brien [58], and Wolden et al. (2000) [37] (single USA institute); and Swerdlow et al. [36], and Swerdlow et al. [62] for the British National Lymphoma Investigation.

Tables 1 and 2 show the abstracted and computed data of the included studies. Overlapping studies were shaded together. The data sets included patients diagnosed as early as 1935 to the late 2000s. The median duration of follow-up since the diagnosis of HL was 14.9 years (95% CI, 13.0-16.2 years; ranging from 6 to 23.8 years), while the median age at the diagnosis of HL was 23.7 years (95% CI, 18.9-25.5 years; ranging from 11.8 to 40 years), and the median age at the diagnosis of SBC was 35.0 years (95% CI, 30.2-40.0 years; ranging from 12.8 to 44.4 years). The median interval between primary diagnosis and development of SBC was 17.7 years (95% CI, 15.2-18.6 years; ranging from 12.2 to 21.6 years).

**Table 1:** Country, study period, design, source of data, study quality, and Hodgkin’s lymphoma therapy details of the 34 studies included in the meta-analysis

**Author & Year**	**Country**	**Year**	**Design**	**Source of data**	**STROBE criteria (met/applicable)**	**RT only No. (%)**	**CTX only No. (%)**	**RT-CTX No. (%)**	**Comments**
Coleman 1987 [23]	UK	1961-1980	PBCS.	Cancer registry	25/32	(35–36)	(20–28)	(27–34)	Therapy data for M + F HL patients. Number NR.
Hancock 1993 [24]	USA	1961–1989	Single institution cohort study	Computerized database and patient records	24/31	383 (43)	30 (3)	341 (39)	Therapy data for M + F HL patients. 15% did not receive any treatment.
Wolden 1998 [30]	USA	1960-1995	Single institution cohort study (HL Dx. ≤21y)	Computerized database and patient records	22/31	144 (47)	9 (3)	154 (50)	Therapy data for female patients
Wolden 2000 [37]	USA	1960-1997	Single institution retrospective review	Computerized database and patient records	24/32	37 (57)	1 (2)	27 (41)	Therapy data for SBC patients. 27 patients received alkylating CTX.
O'Brien 2010 [58]	USA	1970-1990	Single institution study of children	Retrospective chart review and patient questionnaires	23/31	0 (0)	0 (0)	35 (100)	Therapy data for M + F pediatric HL patients. All received low-dose RT and alkylating CTX.
van Leeuwen 1994 [25]	Netherlands	1966-1986	2 institutions cohort study	Institutional registries and patient records	22/30	552 (29)	178 (9)	1209 (62)	Therapy data for M + F HL patients. All SBC received RT.
De Bruin 2009 [55]	Netherlands	1965-1995	6 institutions cohort study (5y survivors)	Medical records, and physician questionnaires	25/32	357 (31.8)	80 (7.1)	685 (61.1)	Therapy data for all HL female patients
Mauch 1996 [26]	USA	1969-1988	5 institutions cohort study	Institutional records	25/31	489 (62)	0 (0)	305 (38)	Therapy data for M + F HL patients
Ng 2002 [41]	USA	1969–1997	4 institutions cohort study	Institutional records	26/32	665 (69)	0 (0)	296 (31)	Therapy data for M + F HL patients
Sankila 1996 [27]	Nordic countries	1940-1987	5 Nordic PBCS (HL Dx. ≤20y)	National cancer registries	21/31	NR	NR	NR	All SBC patients received RT
Metayer 2000 [36]	USA and Europe	1935–1994	16 PBCS (HL Dx. ≤21y, 1-year survivors)	National cancer registries	22/32	NR	NR	NR	
Hodgson 2007 [48]	USA and Europe	1970-2001	13 PBCS (5-year survivors)	National cancer registries	22/32	6461 (34) 145 (74)	4398 (23) 26 (13)	2847 (15) 36 (18)	First raw: therapy data for M + F HL patients. (27% with unknown treatment). Second raw:therapy data for SBC patients (28% with unknown treatment)
Dores 2010 [57]	USA	1973-2000	9 PBCS (5-year survivors, Dx. ≤35y)	9 cancer registry areas of SEER	22/32	NR	NR	NR	All patients had RT
Aisenberg 1997 [28]	USA	1964-1984	Single institution cohort study	Review of patient records	21/31	10 (71)	0 (0)	4 (29)	Therapy data for SBC patients (4 patients had alkylating CTX)
Alm El-Din 2009 [54]	USA	1964-2001	Single institution cohort study	Review of patient records	21/31	130 (52) 24 (67)	0 (0) 0 (0)	118 (48) 12 (33)	First raw: therapy data for all patients (26% had alkylating CTX) Second raw: therapy data for SBC (22% had alkylating CTX)
Hudson 1998 [29]	USA	1968-1990	Single institution	Review of patient records	21/30	116 (30)	15 (4)	256 (66)	Therapy data of M + F HL patients
Gervais-Fagnou 1999 [31]	Canada	1965-1990	Single institution cohort study (HL Dx. at ≤30y)	Review of patient records	22/30	225 (55)	0 (0)	186 (45)	Therapy data of M + F HL patients
Munker 1999 [32]	Germany	1974-1994	6 institutions cohort study	Munich tumor registry, patient records, and patient & family contact	24/31	484 (43.1)	169 (15.1)	464 (41.4)	Therapy data of M + F HL patients (8/9 SBC patients received RT)
Green 2000 [34]	USA	1960-1989	Single institution cohort study (HL ≤20y at Dx.)	Patient records and mail contact	24/30	1 (25)	0 (0)	3 (75)	Therapy data for SBC patients
Swerdlow 2000 [36]	United Kingdom	1963-1993	BNLI (cohort study)	BNLI + 2 cancer databases	24/31	1449 (27)	1693 (31)	2327 (42)	Therapy data of M + F HL patients (all SBC had RT)
Swerdlow 2011 [62]	United Kingdom	1963-2001	BNLI (cohort study)	BNLI database (70 institutions)	25/31	0 (0)	2366 (41)	3432 (59)	Therapy data of M + F HL patients (SBC: 14% CTX only, 86% CTX + RT)
Cellai 2001 [38]	Italy	1960-1991	Single institution cohort study	Institutional patient records	22/30	546 (36)	325 (21)	653 (43)	Therapy data of M + F HL patients (SBC patients: 6% RT, 94% RT + CTX)
Neglia 2001 [39]	USA and Canada	1970-1986	25 institutions (CCSS) cohort study (≤21y at HL Dx., 5-y survivors)	Institutional patient records	26/32	NR	NR	NR	Therapy data were reported for all children malignancies combined
Kenney 2004 [45]	USA and Canada	1970-1986	Same as Neglia et al. [40]	Institutional patient records	26/32	NR	NR	NR	Therapy data were reported for all children malignancies combined
Castellino 2011 [59]	USA and Canada	1970-1986	Same as Neglia et al. [40] and Kenny et al. [46]	Institutional patient records	26/32	263 (33)	46 (6)	472 (60)	Therapy data for all HL female patients
Foss Abrahamsen 2002 [40]	Norway	1968-1985	Single institution cohort study (HD ≥ 1y survivors)	National cancer registry	21/30	447 (44)	202 (20)	363 (36)	Therapy data of M + F HL patients
Bhatia 2003 [42]	USA and Europe	1955-1986	15 institutions cohort study	Institutional patient records	26/33	314 (23) 14 (47)	106 (8) 0 (0)	960 (69) 15 (53)	First raw: therapy data of M + F HL patients Second raw: therapy data of SBC patients
Wahner-Roedler 2003 [44]	USA	1950-1993	Single institution cohort study	Institutional patient records	23/31	322 (50) 23 (77)	0 (0) 0 (0)	327 (50) 7 (23)	First raw: therapy data of M + F HL patients Second raw: therapy data of SBC patients
Behringer 2004 [60]	Germany	1981-1989	Multi-Institutional cohort study	German HL database	23/31	675 (12.9)	618 (11.8)	3947 (75.3)	Therapy data of M + F HL patients
Guibout 2005 [46]	UK and France	1954-1985	8 institutions cohort study	Institutional patient records	28/32	28 (23)	9 (7)	86 (70)	Therapy data of M + F HL patients
Taylor 2007 [51]	UK	1940-1991	PBCS	National Registry of Childhood Tumors	22/31	121 (37) 7 (44)	63 (20) 0 (0)	138 (43) 9 (56)	First raw: therapy data of female HL patients Second raw: therapy data of SBC patients
Basu 2008 [52] Constine 2008 [53]	USA	1960-1990	5 institutions cohort study (<19y at HL Dx.)	Institutional patient records	22/31	174 (44) 18 (62)	37 (9) 3 (10)	187 (47) 8 (28)	First raw: therapy data of M + F HL patients Second raw: therapy data of SBC patients
Howell 2009 [56]	UK	1965-2008	Cohort from a registry and single institution	Institutional patient records and a registry data	27/31	6 (26)	0 (0)	17 (74)	Therapy data of SBC patients
Inskip 2007 [61]	USA	1973-2002	PBCS (< 18y at Dx)	SEER database	29/31	NR	NR	NR	

*BNLI* British National Lymphoma Investigation, *CCSS* Childhood Cancer Survivors Study, *Dx.* diagnosis, *HL* Hodgkin’s lymphoma, *M + F* males and females, NR not reported or data could not be calculated, *PBCS* population-based cohort study, *RT* radiotherapy that included supra-diaphragmatic irradiation, *SBC* second breast cancer*, SEER* Surveillance Epidemiology and End Results, *STROBE* Strengthening the Reporting of Observational Studies in Epidemiology, *y* year. (studies shaded together represent overlapping data).

**Table 2:** Clinical characteristics of the 34 studies included in the meta-analysis

**Author & Year**	**Country**	**Year**	**Design**	**Source of data**	**STROBE criteria (met/applicable)**	**RT only No. (%)**	**CTX only No. (%)**	**RT-CTX No. (%)**	**Comments**
Coleman 1987 [23]	UK	1961-1980	PBCS.	Cancer registry	25/32	(35–36)	(20–28)	(27–34)	Therapy data for M + F HL patients. Number NR.
Hancock 1993 [24]	USA	1961–1989	Single institution cohort study	Computerized database and patient records	24/31	383 (43)	30 (3)	341 (39)	Therapy data for M + F HL patients. 15% did not receive any treatment.
Wolden 1998 [30]	USA	1960-1995	Single institution cohort study (HL Dx. ≤21y)	Computerized database and patient records	22/31	144 (47)	9 (3)	154 (50)	Therapy data for female patients
Wolden 2000 [37]	USA	1960-1997	Single institution retrospective review	Computerized database and patient records	24/32	37 (57)	1 (2)	27 (41)	Therapy data for SBC patients. 27 patients received alkylating CTX.
O'Brien 2010 [58]	USA	1970-1990	Single institution study of children	Retrospective chart review and patient questionnaires	23/31	0 (0)	0 (0)	35 (100)	Therapy data for M + F pediatric HL patients. All received low-dose RT and alkylating CTX.
van Leeuwen 1994 [25]	Netherlands	1966-1986	2 institutions cohort study	Institutional registries and patient records	22/30	552 (29)	178 (9)	1209 (62)	Therapy data for M + F HL patients. All SBC received RT.
De Bruin 2009 [55]	Netherlands	1965-1995	6 institutions cohort study (5y survivors)	Medical records, and physician questionnaires	25/32	357 (31.8)	80 (7.1)	685 (61.1)	Therapy data for all HL female patients
Mauch 1996 [26]	USA	1969-1988	5 institutions cohort study	Institutional records	25/31	489 (62)	0 (0)	305 (38)	Therapy data for M + F HL patients
Ng 2002 [41]	USA	1969–1997	4 institutions cohort study	Institutional records	26/32	665 (69)	0 (0)	296 (31)	Therapy data for M + F HL patients
Sankila 1996 [27]	Nordic countries	1940-1987	5 Nordic PBCS (HL Dx. ≤20y)	National cancer registries	21/31	NR	NR	NR	All SBC patients received RT
Metayer 2000 [36]	USA and Europe	1935–1994	16 PBCS (HL Dx. ≤21y, 1-year survivors)	National cancer registries	22/32	NR	NR	NR	
Hodgson 2007 [48]	USA and Europe	1970-2001	13 PBCS (5-year survivors)	National cancer registries	22/32	6461 (34) 145 (74)	4398 (23) 26 (13)	2847 (15) 36 (18)	First raw: therapy data for M + F HL patients. (27% with unknown treatment). Second raw:therapy data for SBC patients (28% with unknown treatment)
Dores 2010 [57]	USA	1973-2000	9 PBCS (5-year survivors, Dx. ≤35y)	9 cancer registry areas of SEER	22/32	NR	NR	NR	All patients had RT
Aisenberg 1997 [28]	USA	1964-1984	Single institution cohort study	Review of patient records	21/31	10 (71)	0 (0)	4 (29)	Therapy data for SBC patients (4 patients had alkylating CTX)
Alm El-Din 2009 [54]	USA	1964-2001	Single institution cohort study	Review of patient records	21/31	130 (52) 24 (67)	0 (0) 0 (0)	118 (48) 12 (33)	First raw: therapy data for all patients (26% had alkylating CTX) Second raw: therapy data for SBC (22% had alkylating CTX)
Hudson 1998 [29]	USA	1968-1990	Single institution	Review of patient records	21/30	116 (30)	15 (4)	256 (66)	Therapy data of M + F HL patients
Gervais-Fagnou 1999 [31]	Canada	1965-1990	Single institution cohort study (HL Dx. at ≤30y)	Review of patient records	22/30	225 (55)	0 (0)	186 (45)	Therapy data of M + F HL patients
Munker 1999 [32]	Germany	1974-1994	6 institutions cohort study	Munich tumor registry, patient records, and patient & family contact	24/31	484 (43.1)	169 (15.1)	464 (41.4)	Therapy data of M + F HL patients (8/9 SBC patients received RT)
Green 2000 [34]	USA	1960-1989	Single institution cohort study (HL ≤20y at Dx.)	Patient records and mail contact	24/30	1 (25)	0 (0)	3 (75)	Therapy data for SBC patients
Swerdlow 2000 [36]	United Kingdom	1963-1993	BNLI (cohort study)	BNLI + 2 cancer databases	24/31	1449 (27)	1693 (31)	2327 (42)	Therapy data of M + F HL patients (all SBC had RT)
Swerdlow 2011 [62]	United Kingdom	1963-2001	BNLI (cohort study)	BNLI database (70 institutions)	25/31	0 (0)	2366 (41)	3432 (59)	Therapy data of M + F HL patients (SBC: 14% CTX only, 86% CTX + RT)
Cellai 2001 [38]	Italy	1960-1991	Single institution cohort study	Institutional patient records	22/30	546 (36)	325 (21)	653 (43)	Therapy data of M + F HL patients (SBC patients: 6% RT, 94% RT + CTX)
Neglia 2001 [39]	USA and Canada	1970-1986	25 institutions (CCSS) cohort study (≤21y at HL Dx., 5-y survivors)	Institutional patient records	26/32	NR	NR	NR	Therapy data were reported for all children malignancies combined
Kenney 2004 [45]	USA and Canada	1970-1986	Same as Neglia et al. [40]	Institutional patient records	26/32	NR	NR	NR	Therapy data were reported for all children malignancies combined
Castellino 2011 [59]	USA and Canada	1970-1986	Same as Neglia et al. [40] and Kenny et al. [46]	Institutional patient records	26/32	263 (33)	46 (6)	472 (60)	Therapy data for all HL female patients
Foss Abrahamsen 2002 [40]	Norway	1968-1985	Single institution cohort study (HD ≥ 1y survivors)	National cancer registry	21/30	447 (44)	202 (20)	363 (36)	Therapy data of M + F HL patients
Bhatia 2003 [42]	USA and Europe	1955-1986	15 institutions cohort study	Institutional patient records	26/33	314 (23) 14 (47)	106 (8) 0 (0)	960 (69) 15 (53)	First raw: therapy data of M + F HL patients Second raw: therapy data of SBC patients
Wahner-Roedler 2003 [44]	USA	1950-1993	Single institution cohort study	Institutional patient records	23/31	322 (50) 23 (77)	0 (0) 0 (0)	327 (50) 7 (23)	First raw: therapy data of M + F HL patients Second raw: therapy data of SBC patients
Behringer 2004 [60]	Germany	1981-1989	Multi-Institutional cohort study	German HL database	23/31	675 (12.9)	618 (11.8)	3947 (75.3)	Therapy data of M + F HL patients
Guibout 2005 [46]	UK and France	1954-1985	8 institutions cohort study	Institutional patient records	28/32	28 (23)	9 (7)	86 (70)	Therapy data of M + F HL patients
Taylor 2007 [51]	UK	1940-1991	PBCS	National Registry of Childhood Tumors	22/31	121 (37) 7 (44)	63 (20) 0 (0)	138 (43) 9 (56)	First raw: therapy data of female HL patients Second raw: therapy data of SBC patients
Basu 2008 [52] Constine 2008 [53]	USA	1960-1990	5 institutions cohort study (<19y at HL Dx.)	Institutional patient records	22/31	174 (44) 18 (62)	37 (9) 3 (10)	187 (47) 8 (28)	First raw: therapy data of M + F HL patients Second raw: therapy data of SBC patients
Howell 2009 [56]	UK	1965-2008	Cohort from a registry and single institution	Institutional patient records and a registry data	27/31	6 (26)	0 (0)	17 (74)	Therapy data of SBC patients
Inskip 2007 [61]	USA	1973-2002	PBCS (< 18y at Dx)	SEER database	29/31	NR	NR	NR	

*Absolute excess rate of SBC incidence cases per 10,000 person-years of follow-up.

*CI* confidence interval, *Cum.* Cumulative, *DCIS* ductal carcinoma in-situ, *HL* Hodgkin’s lymphoma, *MF* males and females, *NR* not reported or data could not be calculated, *O/E* observed/expected, *RR* relative risk, *SBC* second breast cancer (unless indicated, all are invasive breast cancer), *y* year. (Studies shaded together represent overlapping data).

After eliminating overlapping and duplicate studies, there were 25,305 women with HL and 957 incidences of SBC. The median absolute excess rate (AER) of SBC incidence per 10,000 person-years of observation was 22.9 excess cases (95%CI, 15.6-55.7, ranging from 1.1 to 174). Few studies reported the cumulative incidence of SBC after 20 and 30 years of follow-up (average: 5.4% and 12%, respectively).

### Pooled RR

**Figure 2:**
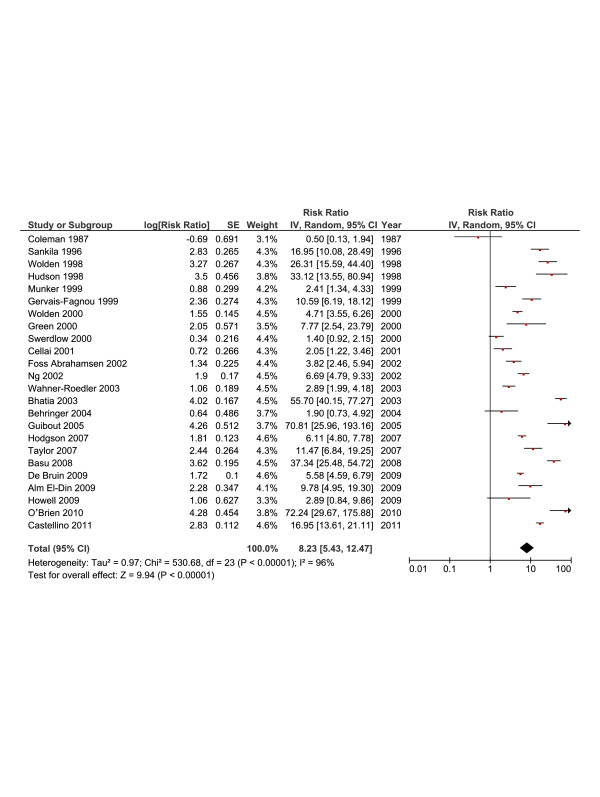
**Summary statistics and corresponding forest plot for the overall relative risk (*RR*) of second breast cancer as reported from 23 studies.**

Figure 2 depicts the Forest plot for the pooled RR. The fixed-effects model showed significant heterogeneity (*I*^*2*^ statistic = 96%; p <0.0001). The random-effects model was computed instead and it showed that patients with HL have an almost 9-fold increase in the risk of SBC (RR = 8.23; [95% CI, 5.43-12.47], *I*^*2*^ = 96%). We performed meta-regression analysis to determine to what extent the heterogeneity is explained by the effects of study size, age at HL diagnosis, and the latency since the completion of HL treatment. The univariate analysis showed significant inverse association between lnRR and age at diagnosis and a positive relation to latency since HL treatment. With multivariate analysis (Table 3), only younger age at diagnosis remained significant (<0.0001).

**Table 3:** The results of meta-regression analyses

**Model**	**Covariates**	**Meta-regression β coefficient (SE)**	**95% CI of β coefficient**	***p*value**
Pooled RR for included studies (Figure 2)	Age at HL diagnosis Latency since HL treatment	−0.105 (0.015) −0.010 (0.031)	−0.137 to −0.072 −0.075 to 0.055	<0.0001 0.747
RR versus follow-up intervals (Figure 6)	Age at HL diagnosis	−0.036 (0.010)	−0.057 to −0.015	0.001
RR versus therapy modality (Figure 7): RT vs. RT + C vs. C	Age at HL diagnosis Latency since HL treatment	−0.099 (0.027) −0.095 (0.073)	−0.157 to −0.041 −0.251 to 0.061	0.003 0.212

*C* chemotherapy, *CI* confidence interval, *HL* Hodgkin’s lymphoma, *RR* relative risk, *RT* radiotherapy, *SE* standard error.

**Figure 3:**
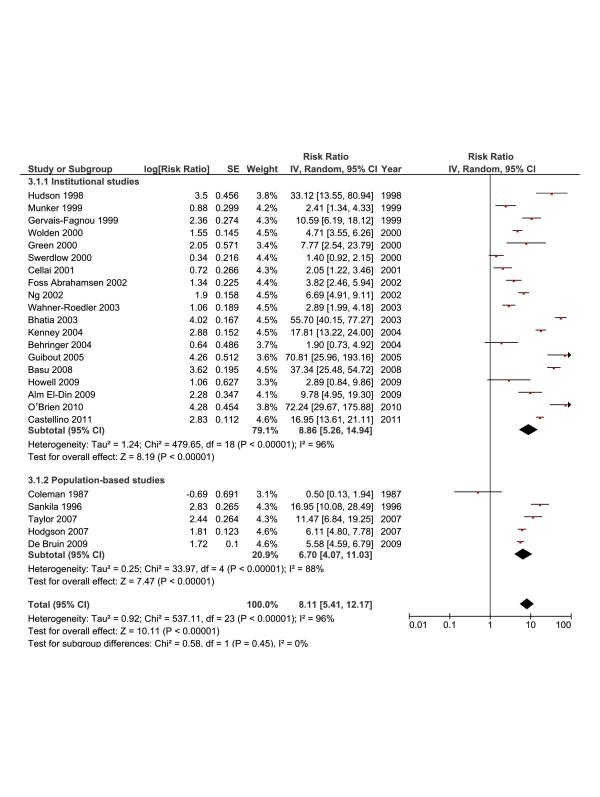
**Summary statistics and corresponding forest plot for the relative risk (*RR*) of second breast cancer in institutional vs. population-based studies.** RRs were calculated using a random- effects model.

In Figure 3, the random-effects analysis illustrates a higher risk in institutional studies (RR = 8.86; [95% CI, 5.26-14.94] compared with population-based analyses (6.70; [95% CI, 4.07-11.03]). The demonstrated heterogeneity (*I*^*2*^ = 96%) was explored by meta-regression analysis, however, none of variables tested was found associated with lnRR in the univariate analysis (data not shown).

### RR and age at diagnosis of HL

**Figure 4:**
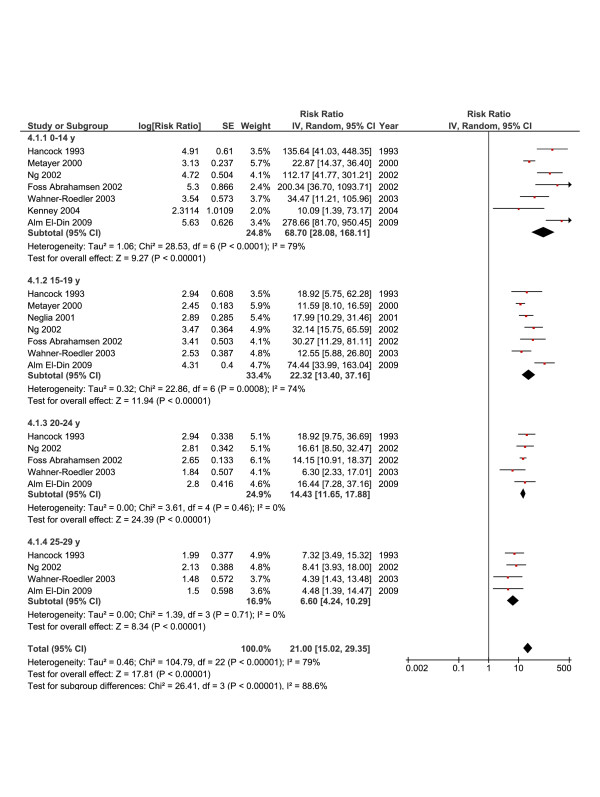
**Summary statistics and corresponding forest plot for the relative risk (*RR*) of second breast cancer based on the age at Hodgkin’s lymphoma diagnosis.** RRs were calculated using a random-effects model.

The excess risk of SBC as a function of age at diagnosis of HL was also explored. Figure 4 (random-effects model) shows that the RR of developing SBC decreased significantly with increasing age at diagnosis from 68.7 (95% CI, 28.8-168.11, ≤ 15 years old) to 22.32 (95% CI, 13.4-37.16, 15–19 years old), 14.43 (95% CI, 11.65-17.88, 20–24 years old), and 6.6 (95% CI, 4.24-10.29, 25–29 years old). As a significant heterogeneity was shown (*I*^*2*^ = 79%), we performed a meta-regression analysis, however, none of the explanatory variables was found significant (data not shown).

**Figure 5:**
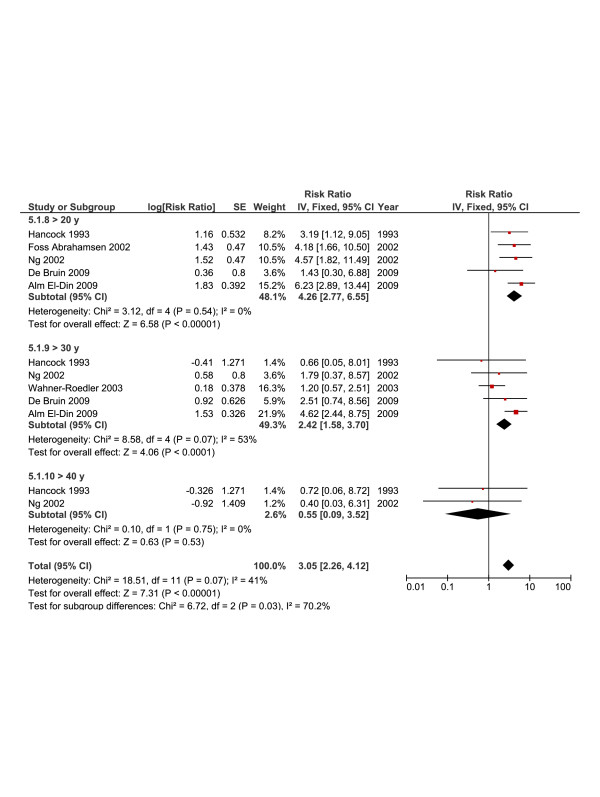
**Summary statistics and corresponding forest plot for the relative risk (*RR*) of second breast cancer based on specific cut points of age at Hodgkin’s lymphoma diagnosis.** RRs were calculated using a fixed-effects model.

Several studies reported the RR at certain cut points of age at diagnosis and it also showed an inverse relation between risk and age (Figure 5, fixed-effects model). In the latter analysis and contrary to analysis of RR against more age intervals, no significant heterogeneity was noted (*I*^*2*^ = 41%). Of note, the RR of SBC for women who developed HL above the age of 40 years was not significant (RR = 0.55; [95% CI, 0.09-3.52]).

### RR and follow-up latency

**Figure 6:**
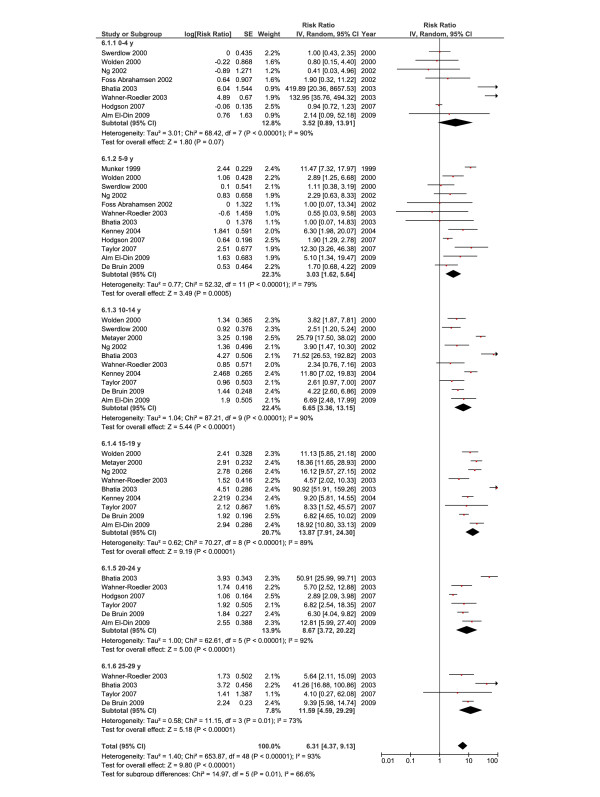
**Summary statistics and corresponding forest plot for the relative risk (*RR*) of second breast cancer based on follow-up latency since Hodgkin’s lymphoma diagnosis.** RRs were calculated using a random-effects model.

We performed analysis of RR by 5-year increments since the treatment of HL (Figure 6, random-effects model, *I*^*2*^ = 73%). By and large, the analysis demonstrated an increasing RR by increased duration of follow-up latency reaching the highest after 15–19 years (RR = 13.87; [95% CI, 7.91-24.30]). While there was a decrease in RR noted after 20–24 years of follow-up, further rise occurred after 25–29 years. The latter rise may be attributed to the RR reported by De Bruin et al. [55], while all the other studies demonstrated a decreased RR after 25–29 years compared with that after 20–24 years of follow-up. Due to unreported data, meta-regression analysis of the heterogeneity could only include age at diagnosis of HL as the sole explanatory variable and it showed an inverse association with lnRR (Table 3).

After ≥ 20 years of latency since diagnosis, 9 studies [37,40,41,44,45,48,51,54,55] reported RR of 6.95 (95% CI, 4.8- 10.1). That RR was not significantly different from the rate encountered after ≥ 30 years of follow-up (RR = 7.03; [95% CI, 5.2-9.5]) as reported from 5 studies [42,44,51,54,55].

### RR vs. Age at HL diagnosis and follow-up latency

To examine the interaction of both age at HL diagnosis and length of follow-up versus risk, few studies have reported adequate data. De Bruin et al. [55] reported that after 5–14 years of follow-up, those who were ≤ 20 years at HL diagnosis had significantly higher risk (RR = 20.0; [95% CI, 7.3-43.4]) as compared with those who were older (21–30 years old) (RR = 5.3; [95% CI, 1.9- 16.6]). Similarly, after ≥ 25 years of follow-up, the corresponding RRs for younger and older patients were 14.2 (95% CI, 7.9-25.4), and 9.0 (95% CI, 4.9-16.5), respectively.

### RR and treatment modalities

**Figure 7:**
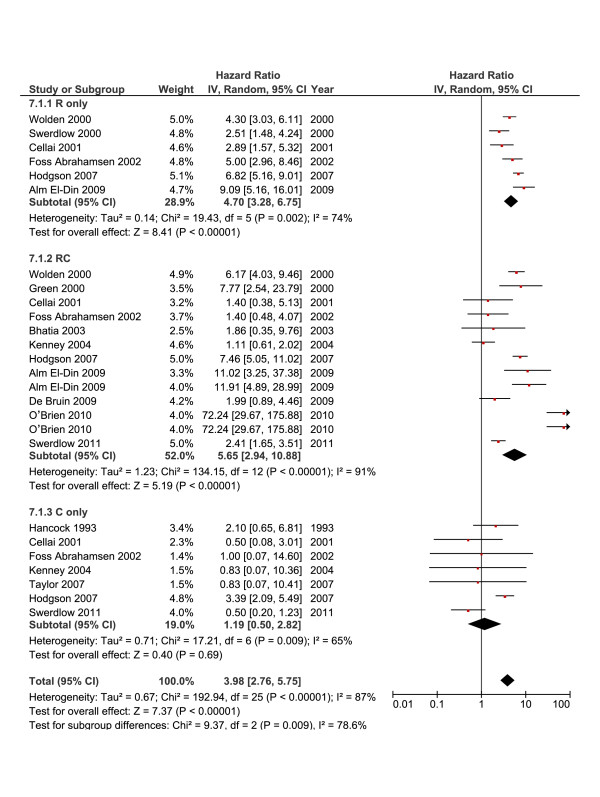
**Summary statistics and corresponding forest plot for the relative risk (*RR*) of second breast cancer vs. treatment modalities.** RRs were calculated using a random-effects model. (*R* radiotherapy, *C* chemotherapy, *RC* combined radiotherapy and chemotherapy).

Figure 7 shows the random-effects model for the RR according to HL treatment modalities. Significant heterogeneity was demonstrated (*I*^*2*^ = 87%). RT used as the sole therapeutic modality was associated with an almost 5-fold increase in risk (RR = 4.70; [95% CI, 3.28-6.75]), *I*^*2*^ = 74%) and even higher rate (RR = 14.08; [95% CI, 9.93-19.98]) when RT was used for patients ≤ 30 years of age [32,36,41]. Two studies [24,54], reported on the mantle field RT dose where there was a small difference in RR between dose < 40 Gy and ≥ 40 Gy (5.99, and 6.13, respectively). In the first study [24], 1 patient per 567 person-years risk versus 23 patients per 7876 person-years developed SBC in the lower versus higher RT dose, respectively. In the second study, 17 of 135 versus 18 of 109 patients developed SBC in the lower versus higher RT dose, respectively [54]. When reported, almost all SBC arose within or at the margin of RT field.

Figure 7 also shows that adding any CT to RT numerically increased the risk as compared with the risk associated with RT only (RR = 5.65; [95%CI, 2.94-10.88], *I*^*2*^ = 91%). Nevertheless, adding alkylating CT to RT did not abate SBC risk (RR = 6.59; [95% CI, 1.72-25.20), while the combination of RT and non-alkylating CT caused a non-significant effect (RR = 4.40; [95% CI, 0.83-23.38]) (data not shown). Noteworthy, only a few studies have provided enough data about the nature of CT offered. The current meta-analysis showed that the use of CT only was not associated with significant risk (RR = 1.19; [95% CI, 0.50-2.82], *I*^*2*^ = 87%).

To explore the heterogeneity of the RR vs. therapeutic modalities, we performed a univariate analysis that showed an inverse association between lnRR and age at HL diagnosis, positive interaction with latency, and no significant effect for study size. The multivariate meta-regression analysis, however, only showed that younger age at diagnosis retained a significant independent risk (Table 3).

### RR and additional contributing factors

Comparing HL patients diagnosed from 1960s to the early 1970s, late 1970s to early 1980s, and more recent years the reported RRs were 3.7, 5.9, and 10.7, respectively [23,28,48,51,55].

Table 4 shows data related to potential contributing factors. Pooled analysis was not attempted due to the small number of studies with sufficient data. Table 4 shows that HL patients who presented with mediastinal mass had higher risk compared with those without mediastinal mass. Table 4 also shows the inconsistency of the reported RR among those who had splenectomy versus those who did no, however, the findings were based on three studies only. Table 4 also shows the potential protective role of pelvic RT as reported by De Bruin et al. [55], where patients receiving that modality showed a risk that was not as high compared with those who did not. HL survivors who received RT had a higher risk of developing estrogen receptor (ER)-negative/progesterone receptor (PR)-negative SBC as compared with ER-positive/PR-positive tumors [57].

**Table 4:** Relative risk of second breast cancer vs. selected risk variables

**Variable**	**RR (95% CI)**
Mediastinal mass +	
Wahner-Roedler 2003 [44]	4.22 (2.71, 6.57)
Alm El-Din 2009 [54]	11.46 (6.78, 19.38)
Mediastinal mass -	
Wahner-Roedler 2003 [44]	1.70 (0.79, 3.63)
Alm El-Din 2009 [54]	6.09 (2.37, 15.67)
Splenectomy +	
Wolden 1998 [30]	2.10 (0.62, 7.16)
Wahner-Roedler 2003 [44]	4.70 (2.87, 7.69)
Alm El-Din 2009 [54]	9.86 (5.42, 17.92)
Splenectomy -	
Wahner-Roedler 2003 [44]	1.90 (1.05, 3.45)
Alm El-Din 2009 [54]	9.67 (4.87, 19.20)
Mantle RT, no pelvic RT	
De Bruin 2009 [55]	8.20 (6.62, 10.15)
Mediastinal RT, no pelvic RT	
De Bruin 2009 [55]	3.71 (1.38, 9.97)
Mantle RT + pelvic RT	
De Bruin 2009 [55]	2.70 (1.11, 6.56)
ER-/PR-	
Dores 2010 [57]	9.30 (7.00, 12.36)
ER+/PR+	
Dores 2010 [57]	4.95 (3.84, 6.39)

*CI* confidence interval, *ER* estrogen receptors, *PR* progesterone receptors, *RR* relative risk, *RT* radiotherapy.

## Discussion

HL has been a successful model for the development of effective treatment approach in clinical oncology. Long-term survivors of that disease have also allowed better recognition and understanding of the late effects of therapy. In a large cohort of 25,305 women with HL, and with 957 incidences of SBC reported from North American and European institutions, the current meta-analysis quantified the risk of SBC. The overall pooled analysis showed that there was an approximate 9-fold increase in the risk of SBC incidence (pooled RR = 8.23), and AER of 23 patients per 10,000 person-years.

Patients included in this meta-analysis developed HD at a median age of 23.7 years. There has been incongruity about the influence of younger age at HL diagnosis and the higher risk of SBC, where some studies have failed to prove that effect [39,42]. The current meta-analysis clearly showed that younger age at HL diagnosis was associated with increased risk of SBC, and the risk remained after adjusting for other covariates. Moreover, we demonstrated that the RR of SBC for women who developed HL above the age of 40 years was not significant. It is presumed that the higher risk associated with young age at HD diagnosis is attributed to the effect of RT delivered at a time when breast tissue is proliferating.

RT used as the sole therapeutic modality was associated with a 5-fold (RR = 4.70) increase in risk and a 14-fold (RR = 14.08) increase among young (≤ 30 years of age). Almost all SBC arose within or at the margin of the RT field. The RR of combined RT and any CT was slightly higher than that associated with RT only (5.65 vs. 4.70). An even higher, was the RR associated with the combination of RT and alkylating CT (RR = 6.59), thus, the potential protective effect of gonado-toxic alkylating CT was not demonstrated. Several studies showed an inverse association between the use of alkylating CT in HL and SBC risk [24,65], however, other investigators reported increased risk [30,66]. In this meta-analysis and based on data reported from three studies, the risk associated with the combination of RT and non-alkylating CT was not significant [37,54,58], also found insignificant, was the risk related to the use of CT only.

Analysis of potential additional risk factors was limited due to lack of sufficient data and/or inability to compute missing information, therefore, cautions should be exerted in interpreting results. Two studies [24,54], reported on the mantle field RT dose where there was only a small difference in RR between dose < 40 Gy and ≥ 40 Gy (5.99, and 6.13, respectively). While some studies showed that subjects with SBC were found to be significantly more likely to have received higher doses of mantle RT [52], this observation was complicated by the fact that patients treated with higher radiation doses have had longer follow-up. Guibout et al. [46], did not find a significant association between RT dose and SBC, suggesting that the increased risk after HL may indicate a specific susceptibility for developing SBC, or a particular susceptibility to radiation and/or chemotherapy, or both. Conversely, De Bruin et al. showed that the risk of SBC is related to the RT volume [55], where mantle field irradiation was associated with a 2.7-fold increased risk of SBC compared with mediastinal irradiation alone. Besides, the meta-analysis reported by Franklin et al. showed a RR of 3 comparing extended field versus involved field RT [14].

The reason for failing to show a convincing evidence of RT dose–response effect associated with SBC risk is at best divisive. There is evidence for a strongly linear radiation dose response, but only in the lower dose range (up to 5 or 10 Gy) [67,68]. It has been suggested that cell killing tends to decrease the carcinogenic effect of RT along an exponential curve at doses above 10 Gy [69]. However, it is known that BC is a known complication of low-dose breast radiation [67], thus BC may remain a risk among adolescent women who receive any dose of thoracic irradiation for HL.

Although new RT techniques and treatment strategies have the potential to reduce the future burden of late effects, nevertheless, we have shown that an even higher risk was reported in more recent years suggesting that there remains a significant cohort at an increased risk of SBC.

Pelvic RT was found to be associated with a protective effect as reported by De Bruin et al. [55]. The same effect was also noted by Basu et al., where 3.4% of patients who developed SBC received pelvic RT as compared with 26.3% among those who did not [52]. It is presumed that the protective effect of pelvic RT is attributed to the induction of premature menopause and the role played by hormone stimulation in RT-induced breast carcinogenesis [43,70]. The influence of splenectomy on SBC risk has been controversial. While some studies reported a modest higher risk [71], other studies failed to show that effect [30,52].

Only one study examined the receptor status of SBC [57]. The RR of ER-negative/PR-negative SBC was 66% higher than ER-positive/PR-positive SBC among 5-year HL survivors, and nearly two-fold higher among 15-year survivors. Conversely, other studies of small numbers of SBC patients have not found a significant variation in hormone-receptor status when compared with primary BC controls [72,73]. While the incidence of hormone receptor-positive BC in the general population exceeds that of ER-negative/PR-negative BC, it is postulated, however, that young women treated for HL may experience premature ovarian failure related to HL therapy, and therefore, their hormonal BC risk factors may differ from those in the general population.

The present meta-analysis has several limitations. First, it is not possible to completely exclude the possibility that the HL itself carries with it an increased risk of second malignancy including SBC. Second, it is very difficult to quantify the possible effect of confounding factors such as lifestyle factors, personal risk, family history, etc. For example, Landgren et al. found increased RR (1.81) of breast cancer among HL patient with positive (vs. negative) family history of cancer [50]. Third, the analyses showed significant heterogeneity in risk estimate, nevertheless, investigating heterogeneity using meta-regression technique showed the dominant role of age at HL diagnosis. Other limitations include the lack of comprehensive treatment data including information on RT dose and additional treatments, and the lack of sufficient data to model the protective effect of endogenous hormone ablation against the risk associated with exposure to exogenous hormones. Moreover, it is not clear if a similar magnitude of risk is to be expected in a different patient population where the incidence of sporadic BC is low. Finally, the meta-analysis lacks the analysis of SBC outcome. However, SBC incidence rather its mortality was the main objective of the meta-analysis. Moreover, not all studies reported on mortality, besides, analysis of SBC mortality would be confounded by the mortality from HL itself or its therapy-related effect, ascertainment of the cause of death, age of diagnosis of HL or SBC, and length of follow-up.

## Conclusions

We conclude that the current meta-analysis provided the most recent comprehensive estimate of the risk of SBC in a broad-range of HL survivors with inclusive analysis of relevant clinical and treatment variables. Based on the derived data where the median age at the diagnosis of SBC was 35.0 years and at a median latency of 17.7 years, screening recommendations for HL survivors need to be reemphasized. The results from the current meta-analysis support the favorable outcome of the risk-guided BC screening for such patients according to three prospective studies [74-76]. It is probably more appropriate that female patients with HL who are at a higher risk for developing SBC to be screened annually and at an earlier age rather than biennially starting at the age of 50 years as currently recommended for general population [77]. Our data also support the recent trend of risk-adapted management of HL to reduce the risk of short- and long-term adverse events associated with needless overtreatment [78].

## Competing interests

All authors declare that they have no competing interests.

## Authors’ contributions

EMI, KMA, GAK, OAA, and MA Conception and design of the meta-analysis. EMI Study coordination and tasks’ assignment. KMA, and GAK Initial literature search. EMI, KMA, GAK, OAA, and MA Review of all potential studies. EMI, KMA, GAK, OAA, and MA Data extraction. EMI, KMA, GAK, OAA, and MA Assessing quality of included studies. EMI Statistical analysis. EMI, KMA, and GAK Investigating heterogeneity. EMI, KMA, GAK, OAA, and MA Preparation of the manuscript. EMI, KMA, GAK, OAA, and MA Reading the final manuscript. EMI, KMA, GAK, OAA, and MA Approval of the final manuscript. All authors read and approved the final manuscript.

## Pre-publication history

The pre-publication history for this paper can be accessed here:

http://www.biomedcentral.com/1471-2407/12/197/prepub
